# Psychometric properties of the Japanese version of the Recovery Attitudes Questionnaire (RAQ) among mental health providers: a questionnaire survey

**DOI:** 10.1186/s12888-016-0740-x

**Published:** 2016-02-16

**Authors:** Rie Chiba, Maki Umeda, Kyohei Goto, Yuki Miyamoto, Sosei Yamaguchi, Norito Kawakami

**Affiliations:** School of Nursing, Jichi Medical University, 3311-159, Yakushiji, Shimotsuke-shi, Tochigi 329-0498 Japan; Faculty of Community Health Nursing/ Public Health Nursing, St Luke’s, International University, 10-1, Akashi-cho, Chuo-ku, Tokyo, 104-0044 Japan; Tokyo Musashino Hospital, 4-11-11, Komone, Itabashi-ku, Tokyo, 173-0037 Japan; Department of Psychiatric Nursing, Graduate School of Medicine, The University of Tokyo, 7-3-1, Hongo, Bunkyo-ku, Tokyo, 113-0033 Japan; Department of Psychiatric Rehabilitation, Institute of Mental Health, National Center of Neurology and Psychiatry, 4-1-1, Ogawahigashi-cho, Kodaira-shi, Tokyo, 187-8553 Japan; Department of Mental Health, Graduate School of Medicine, The University of Tokyo, 7-3-1, Hongo, Bunkyo-ku, Tokyo, 113-0033 Japan

## Abstract

**Background:**

“Recovery” is a central concept in mental health, particularly for mental health services and policy-makers. The present study examined the factorial and concurrent validity, internal consistency reliability, and test–retest reliability of the Japanese version of the 7-item Recovery Attitudes Questionnaire (RAQ) among mental health service providers in community and inpatient settings in Japan.

**Methods:**

We conducted a cross-sectional questionnaire with a number of eligible professional groups, including psychiatrists, registered/assistant nurses, public health nurses, clinical psychologists, pharmacists, occupational therapists, and social workers. Participants were drawn from two psychiatric hospitals and 56 psychiatric clinics or community service agencies. In total, 331 participants completed the questionnaire. After excluding those with missing RAQ values, 307 participants were included in the analysis; the participants’ mean age was 40.2 years and 29.6 % were men. The questionnaire comprised the Japanese version of the 7-item RAQ developed by the present authors, the revised scale of the positive attitudes of staff toward persons with mental disorder (the positive attitudes scale), and the Japanese-language version of the Social Distance Scale (SDSJ). Confirmatory factor analyses were used to examine factorial validity of a two-factor structure reported in a previous study (Borkin et al., 2000) as well as a single-factor structure. Concurrent validity was determined by calculating correlations between RAQ and the other two scales. Internal consistency reliability was assessed with Cronbach’s alpha coefficients and inter-item correlations. Test–retest reliability was assessed by the intraclass correlation coefficient (ICC), with a weighted kappa in a subsample of participants (*n* = 13).

**Results:**

The two-factor structure showed acceptable factorial validity. RAQ scores were significantly and positively correlated with the positive attitudes scale, and there was a significant inverse correlation with the SDSJ (*p* < 0.01). The RAQ had an overall Cronbach’s alpha coefficient of 0.64. Four inter-item correlations were not significant. The ICC and weighted kappa values indicated unsatisfactory test–retest reliability.

**Conclusion:**

The Japanese RAQ showed acceptable factorial validity, reasonable concurrent validity, and unsatisfactory reliability in community and inpatient mental health settings in Japan. Further large-scale research is required to ensure robust verification.

**Electronic supplementary material:**

The online version of this article (doi:10.1186/s12888-016-0740-x) contains supplementary material, which is available to authorized users.

## Background

“Recovery” is a central concept in mental health, particularly for mental health services and policy-makers. The recovery concept first emerged in the 1980s as a movement led by people with mental illness in the US. Since then, the recovery concept has prevailed in Western countries and more recently, in other parts of the world such as Asian and African countries [1–4]. Recovery is defined as a complex process of developing new meaning and purpose in life as one grows beyond the catastrophic effects of mental illness [5]. Recovery is considered to be an individualized process related to subjective, multifaceted dimensions such as hope, self-identity, meaning in life, and personal responsibility [6]. This concept clearly differs from the traditional treatment-based concept of recovery, which focuses on the illness with a goal of symptomatic remission or functional improvement.

Even though recovery can be achieved without mental health services, mental health service providers play an important role in facilitating recovery, as they provide professional, directive support [7, 8]. Embracing recovery requires mental health service providers to approach service provision with new way of thinking, refined skills, and attitudes that include developing relationships within an even power balance [9]. For example, it is necessary to develop self-control of own anxiety when people with mental illness would take risks in trying something new, and acknowledging that people with mental illness have expertise through experience [10]. Embracing recovery also includes emphasizing an individual’s possibilities, rather than concentrating on the problems; this means that service providers support people with mental illness to identify their own strengths [11, 12]. Service providers’ attitudes toward recovery also help to foster a recovery-oriented organizational system that goes beyond individual-level care provision [13].

Several studies in Western populations have suggested that service providers’ attitudes and knowledge about the several aspects of recovery can be improved [9, 11]. Service providers’ attitudes toward recovery are considered to be associated with positive attitudes toward people with mental illness. Other studies have found that some service providers exhibit harmful stigma toward people with mental illness that may impede recovery-oriented care [14–17]. This stigma toward them is considered to be negatively associated with service providers’ attitudes toward recovery.

To date, various scales have been developed to assess an individual’s attitude toward recovery or knowledge of recovery [8, 18, 19]. Among these, the Recovery Attitudes Questionnaire (RAQ) [19], developed in the US, is a representative scale that assesses attitudes about the belief that people can recover from mental illness. Borkin et al. (2000) theoretically established a two-factor structure for RAQ items, and found marginally adequate internal consistency in a US sample [19]. Subsequent studies with Australian and Dutch mental health professionals examining the psychometric properties of RAQ suggested further research was necessary to confirm the suitability of RAQ in those countries [20, 21]. This suggests that elaborative evaluation is needed to consider the cross-cultural applicability of RAQ.

To enhance recovery among people with mental illness, many programs have been developed for people with mental illness and for service providers. RAQ has been used in such interventional studies over the past decade to examine their effectiveness [22–24]. Examining the reliability and validity of the Japanese version of RAQ will be useful to assess its possible use in future studies in Japan. To date, most of the studies on recovery have been conducted in community settings; however, inpatients in psychiatric wards also have their own paths to recovery [25, 26], and recovery-oriented principles should be applied in inpatient settings [27, 28].

The present study aimed to examine the factorial validity, concurrent validity, internal consistency reliability, and test–retest reliability of the Japanese version of the 7-item RAQ among mental health service providers in community and inpatient settings in Japan. We hypothesized that the Japanese version of RAQ would show good factorial validity to the two-factor structure and good reliability. We also hypothesized that RAQ scores would show significant correlation with the scores on related scales, in the assumed direction for each related scale.

## Methods

### Participants

A cross-sectional, self-administered questionnaire survey was conducted with mental health service providers from February to March 2012. Professional groups eligible for inclusion whether they worked full- or part-time were: psychiatrists, registered/assistant nurses, public health nurses, clinical psychologists, pharmacists, occupational therapists, and social workers. We conducted the survey in two psychiatric hospitals in the Kanto region and 56 psychiatric clinics or community service agencies in Tokyo, Japan.

There were 220 eligible professionals in the two psychiatric hospitals; 180 of these agreed to participate and returned completed questionnaires (response rate = 81.8 %). In the psychiatric clinics and community service agencies, there were 255 eligible professionals, and 151 agreed to participate and responded to the questionnaire (response rate = 59.2 %). This gave a total of 331 respondents; 24 were excluded because they were missing responses for one or more RAQ items. We used data from the remaining 307 participants for the analyses.

### Measures

The questionnaire included RAQ, the revised scale of the positive attitudes of staff toward persons with mental disorder (the positive attitudes scale), and the Japanese-language version of the Social Distance Scale (SDSJ). We also collected data on socio-demographic and occupational variables.

### Development of the Japanese version of the 7-item RAQ

RAQ includes seven items such as “Recovery can occur even if symptoms of mental illness are present.” Responses are on a 5-point Likert scale ranging from “strongly disagree” to “strongly agree” [19]. Higher total scores indicate a more positive attitude to the concept of recovery. Two specific RAQ domains were determined with factor analysis: 1) recovery is possible and needs faith, and 2) recovery is difficult and differs among people. A US study demonstrated that the 7-item RAQ has sound internal consistency reliability, test–retest reliability, and factorial validity [19]. RAQ includes a brief introduction before the items:

*“Recovery is a process and experience that we all share. People face the challenge of recovery when they experience the crises of life, such as the death of a loved one, divorce, physical disabilities, and serious mental illnesses. Successful recovery does not change the fact that the experience has occurred, that the effects are still present, and that one’s life has changed forever. Rather, successful recovery means that the person has changed, and that the meaning of these events to the person has also changed. They are no longer the primary focus of the person’s life.”* [5]

We translated the 7-item RAQ into Japanese with the consent of the original developer, referring to relevant guidelines for translating and adapting psychometric scales [29]. We developed the Japanese 7-item RAQ in five steps. 1) Forward translation: two of the present authors independently translated RAQ from English into Japanese. 2) Reconciliation: five mental health researchers discussed and reached consensus on a draft Japanese translation of RAQ that best reflected the literal and conceptual content of the original English version. 3) Cognitive debriefing and review of cognitive debriefing results: two mental health service providers, a peer-support group leader with chronic mental illness, and five peer-support group participants tested RAQ, after which the present authors reworded the items as necessary to ensure they were understandable. 4) Back-translation: two native English-speaking professional translators, who had not seen the original English RAQ back-translated the Japanese version into English. 5) Back-translation review and finalization: the present authors reviewed the back translations against the source instrument to ensure the literal and conceptual equivalence of the translation. The translation included the introductory sentences described above [5]. Additional file 1 shows the Japanese version of the RAQ.

To examine the construct and concurrent validity of RAQ, we used two additional scales to assess staff attitudes toward people with mental illness.

### The positive attitudes scale

The positive attitudes scale is a 19-item scale, comprising three domains covering expectations about the ability and recovery of people with mental illness, attitudes toward living alongside those people, and supportive helping behaviors. The items, for example, “I think most people with mental illness can take responsibility for their own lives” are rated on a 4-point Likert scale, ranging from “strongly disagree” to “strongly agree.” A higher score indicates a more positive attitude toward people with mental illness. The good internal consistency reliability and good convergent validity of the positive attitudes scale have been previously confirmed among mental health service providers in Japan [30].

### SDSJ

SDSJ was developed in reference to the original scale created by Whatley [31], which was a 5-item scale designed to assess an individual’s sense of social distance from people with schizophrenia. SDSJ includes items such as “I think it best not to associate with people with schizophrenia.” Responses are on a 4-point Likert scale ranging from “disagree” to “agree,” with a higher scale score indicating a more negative attitude. SDSJ has been found to have good internal consistency reliability, good test–retest reliability, and acceptable factorial validity [32]. Though RAQ is a scale to assess one’s attitude for not people with schizophrenia but inclusive people with mental illness, we used the unchanged version of SDSJ, because a revised version with confirmed reliability and validity was not available.

### Statistical analysis

Borkin et al. (2000) [19] demonstrated a theoretically significant two-factor structure for RAQ (Factor 1 = “Recovery is possible and needs faith; Factor 2 = “Recovery is difficult and differs among people). We conducted confirmatory factor analysis (CFA) to test the model fit of the data to the factor structure. In addition, as RAQ is a 7-item scale, we used CFA for a single-factor structure. CFA were conducted in AMOS ver. 23.0 using structural equation modeling (SEM). Maximum likelihood estimation was used to examine goodness of fit for the models using the following criteria [33]: goodness of fit index (GFI) >0.90; adjusted goodness of fit index (AGFI) >0.90; comparative fit index (CFI) >0.90; and a smaller Akaike’s information criterion (AIC) that indicates better model. Concurrent validity was assessed by calculating Pearson’s correlation coefficients between the total RAQ score and the scores for the other two scales.

We calculated Cronbach’s alpha coefficients for the total RAQ score, and for each factor score. As Cronbach’s alpha coefficients may not be sufficient to measure the homogeneity of each item [34], we also calculated inter-item correlations. Test–retest reliability was examined by calculating the intraclass correlation coefficient (ICC) for the total score and quadratic weighted kappa values for each item in a subsample of participants (*n* = 13) who were re-surveyed 2 weeks after the original questionnaire. This test–retest time interval was chosen as we considered it was long enough to prevent recall of previous answers, while being short enough to assume that the condition would not change in most cases. The scores were compared with ICC standards (almost perfect, >0.81; substantial, 0.61–0.80; moderate, 0.41–0.60; fair, 0.21–0.40; and slight, 0.0–0.20) [35] and kappa values (good, >0.61; moderate, 0.41–0.60; slight, 0.21–0.40; and poor, <0.20) [36].

All statistical analyses, including descriptive analyses (other than those described above), were conducted using SPSS 23.0 J for Windows. *P* values of less than 0.05 were considered statistically significant (two-tailed tests).

### Ethical considerations

The aim and procedures of this study were approved by the Ethical Committee of The University of Tokyo. It was qualified to approve recruitment at all sites from which participants were enrolled. All participants received full explanations in writing about the purpose and methods, as well as the data storage and privacy protection methods employed in the study. Providing an answer represented their agreement to participate in the study. The survey was conducted on an anonymous basis.

## Results

### Participant characteristics

Table 1 presents participants’ socio-demographic and occupational characteristics. About 70 % of the participants were female, 40 % were registered or assistant nurses, and 35 % were psychiatric social workers. The mean age of participants was 40.2 years (standard deviation [SD] = 11.8 years; range 22–74 years), and the mean length of work experience in psychiatric services was 9.8 years (SD = 8.4 years; range 0–45 years).

**Table 1:** Participants’ socio-demographic and occupational characteristics (*n* = 307)

	*N* = 307	
Variables	*n* [Mean]	(%) [SD]
Sex (male)	91	(29.6)
Age (years)	[40.2]	[11.8]
Years of work tenure in psychiatric or mental health services	[9.8]	[8.4]
Occupation		
Registered nurse/Assistant nurse	134	(43.6)
Social worker	109	(35.5)
Occupational therapist	20	(6.5)
Clinical psychologist	19	(6.2)
Psychiatrist	16	(5.2)
Pharmacist	6	(2.0)
Public health nurse	3	(1.0)
Education		
High school	6	(2.0)
Vocational school	115	(37.5)
Junior college	20	(6.5)
College	130	(42.3)
Graduate school	34	(11.1)
Unknown	2	(0.7)
Employment status		
Full-time job	245	(79.8)
Part-time job	53	(17.3)
Unknown	9	(2.9)
Department		
Ward	131	(42.7)
Out-patient clinic/Home-visit nursing	40	(13.1)
Psychiatric day-care	27	(8.8)
Home assistance/rehabilitation	16	(5.2)
Group home	5	(1.6)
Job assistance	62	(20.2)
Community activity support center	2	(0.7)
Others, Unknown	24	(7.8)

The mean total RAQ score was 27.98 (SD = 2.85; range 14–35). Item 7 showed a marginal ceiling effect; that is, most participants agreed with this item, meaning collective scores were disproportionately higher. The mean RAQ total scores by occupation (in descending order) were: 28.05 (SD = 2.22) for clinical psychologists (n = 19); 27.40 (SD = 2.46) for occupational therapists (*n* = 20); 27.19 (SD = 2.93) for social workers (*n* = 109); 27.00 (SD = 2.48) for psychiatrists (*n* = 16); and 26.63 (SD = 3.04) for registered nurses/assistant nurses (*n* = 134). Total scores were not calculated for pharmacists (*n* = 6) and public health nurses (*n* = 3) as these samples were small. We found no significant differences between the occupations.

**Table 2:** Results of confirmatory factor analysis: comparison of goodness-of-fit indices between one- and two-factor RAQ models (*n* = 307)

Model	GFI	AGFI	CFI	AIC	Chi-square	*df*	*p*
1-factor ^a)^	0.93	0.85	0.77	111.85	83.85	14	0.00
**2-factor** ^**b)**^	**0.95**	**0.90**	**0.86**	**85.26**	**55.26**	**13**	**0.00**

*GFI* Goodness of fit index, *AGFI* Adjusted goodness of fit index, *CFI* Confirmatory fit index, *AIC* Akaike information criterion

df: degrees of freedom; better fit model denoted by bold letters

^a)^ All seven items loaded on one factor

^b)^ Each item loaded on a two-factor structure [19]

### Validity of RAQ

1. Factorial validity
  The two-factor structure [19] fitted the data better than the single-factor structure (Table 2). GFI and AGFI for the two-factor structure indicated a good fit, although CFI did not reach the recommended standard (GFI = 0.95; AGFI = 0.90; CFI = 0.86; AIC = 85.26). However, three of the seven RAQ items had relatively small loadings: Item 2 “To recover requires faith” (0.26), item 5 “Recovering from mental illness is possible no matter what you think may cause it” (0.33), and item 7 “People differ in the way they recover from a mental illness” (0.35) (Fig. 1).

**Fig. 1:**
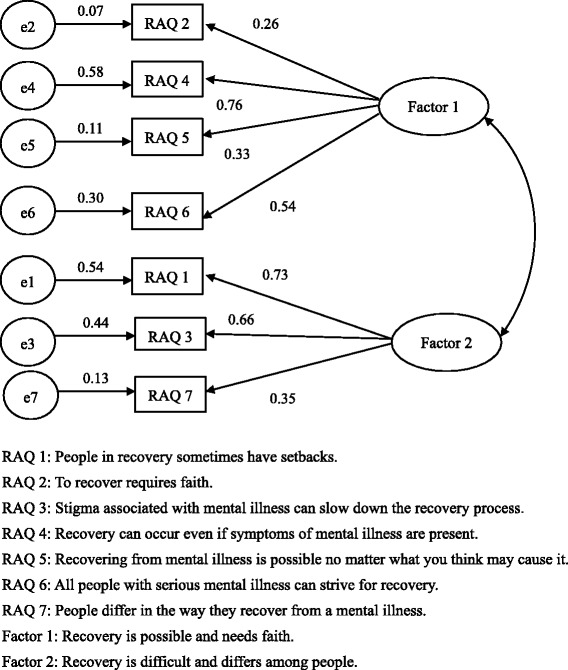
Path diagram of the Japanese version of RAQ, showing standardized coefficients from confirmatory factor analysis
2. Concurrent validity
  The mean total score for the positive attitudes scale was significantly and positively correlated with the mean RAQ total score (*r* = 0.38; *p* < 0.01), the mean RAQ Factor 1 score (*r* = 0.35; *p* < 0.01), and the mean RAQ Factor 2 score (*r* = 0.24; *p* < 0.01). The mean total SDSJ score was significantly and negatively correlated with the mean RAQ total score (*r* = −0.29; *p* < 0.01), the mean RAQ Factor 1 score (*r* = −0.26; *p* < 0.01), and the mean RAQ Factor 2 score (*r* = −0.21; *p* < 0.01).
3. Reliability of RAQ (internal consistency reliability and test–retest reliability)
  Cronbach’s alpha coefficients were 0.64 for the mean total RAQ score, 0.53 for the mean Factor 1 score (Recovery is possible and needs faith, four items), and 0.56 for the mean Factor 2 score (Recovery is difficult and differs among people, three items). In addition, inter-item correlation coefficients were moderate to slight (Table 3). While most correlations were significant, the correlations for RAQ items 1 and 6, items 2 and 5, items 3 and 6, and items 6 and 7 were not significant.

**Table 3:** Correlation matrix between each item on the Japanese version of RAQ (*n* = 307)

	Correlations						
	RAQ 1	RAQ 2	RAQ 3	RAQ 4	RAQ 5	RAQ 6	RAQ 7
RAQ 1	1						
RAQ 2	0.21^**^	1					
RAQ 3	0.22^**^	0.27^**^	1				
RAQ 4	0.38^**^	0.14^*^	0.17^**^	1			
RAQ 5	0.22^**^	0.10	0.21^**^	0.43^**^	1		
RAQ 6	0.11	0.23^**^	0.07	0.21^**^	0.29^**^	1	
RAQ 7	0.49^**^	0.16^**^	0.25^**^	0.36^**^	0.11^*^	0.04	1

^**^ *p* < 0.01

^*^ *p* < 0.05
  ICC for the mean total RAQ score was 0.68, indicating substantial reliability. The weighted kappa values for each item were also examined. Two of the seven items had weighted kappa values of 0.41–0.60, indicating moderate reliability, while the other five items (RAQ items 1, 2, 4, 5, and 6) had weighted kappa values of 0.40 or less, indicating slight or poor reliability.

## Discussion

The Japanese version of the 7-item RAQ showed acceptable factorial validity, reasonable concurrent validity, and unsatisfactory internal consistency reliability and test–retest reliability among mental health professionals in Japan.

CFA showed that the two-factor model [19] fit the data acceptably, though RAQ item 2 (To recover requires faith) had particularly small factor loading (0.26) for Factor 1 (Recovery is possible and needs faith). Given that this item indicates an attitude that recovery is difficult without faith, it may be argued that it also comes under Factor 2 (Recovery is difficult and differs among people). Studies using the Dutch version of RAQ [21] and in Australia [20] also found that RAQ item 2 had lower factor loading and did not fit the factor structure. Faith is a concept with diverse meanings, dependent on the culture, context, and even the setting in which it is used [20, 37]; it describes confidence, belief, or trust in a being, object, living organism, deity, view, or the doctrines or teachings of a religion. In Western or secular culture, faith has been argued as being present in science-based clinical practice, because science has replaced religion [38]. However, in Japan, concepts of faith may include the inner mind and spirituality stemming from traditional Japanese culture that values virtue and loyalty over science. At the same time, the nuances of this interpretation of Japanese faith may vary depending on the translated expression. Therefore, to validate cross-cultural applicability, it will be necessary to define faith in the context of RAQ, and to refine the expression used after a careful review of the wording in the Japanese version.

The positive attitudes score was significantly and positively correlated with the RAQ score, while the SDSJ score was significantly and inversely correlated with the RAQ score. Although these correlations were not strong, they were consistent with our hypotheses. The moderate correlation between the RAQ and the positive attitudes scores suggests that while conceptually not the same, they are close. The RAQ domain, “Recovery is possible,” was similar to the positive attitudes scale domain “An expectation for the ability and recovery of people with mental illness.” On the other hand, the RAQ domain “Recovery is difficult and differs among people” did not overlap with the other two domains of the positive attitudes scale (attitude toward living alongside people with mental illness, and supportive helping behaviors). The significant and inverse correlation between the RAQ and SDSJ scores was consistent with previous studies conducted in the US [39, 40]. As previous conceptual and empirical studies have suggested that the recovery of people with mental illness can be impeded by stigma [41–43], this finding demonstrates the reasonable concurrent validity of the Japanese version of RAQ. A possible reason for the relatively weak correlation observed in our study seems due to the difference in the target diseases between RAQ (mental illness in general) and SDSJ (schizophrenia). This difference may affect the results, since people with schizophrenia are more likely to be stigmatized compared to those with other mental illness [44].

The Cronbach’s alpha coefficients in the present study (0.64, 0.53, and 0.56 for the total RAQ and factor scores) indicated the Japanese RAQ had unsatisfactory internal consistency reliability; although this is limited by the small number of scale items [19], the values were not sufficiently high to meet the recommended 0.72 standard [34]. The relatively modest values we found are comparable to previous studies using RAQ that reported values of 0.57 (German version) [45] and 0.61 (Dutch version) [21]. However, other studies have reported somewhat higher Cronbach’s alpha coefficients, with 0.70 in the US [19], 0.70 for the German version [46], and 0.74 in Australia [20]. Inter-item correlation coefficients showed that RAQ item 6 (All people with serious mental illness can strive for recovery) had insignificant and weak correlations with RAQ items 1 (People in recovery sometimes have setbacks), 3 (Stigma associated with mental illness can slow down the recovery process), and 7 (People differ in the way they recover from a mental illness). As some of our participants worked in inpatient settings and provided care for people with severe mental illness, there might be a tendency to not agree with item 6, regardless of positive answers to the other items. This might explain the poor correlations between those items.

We found the Japanese RAQ had reasonable test–retest reliability by the ICC. However, the quadratic weighted Kappa values for five of the seven items ranged from slight to poor. Although the specific reason for this is unclear, the non-systematic variability cannot be justified in a two-week test–retest interval. These item scores might depend on changing mood or other conditions related to a short time span. One can also argue that even 2 weeks interaction between providers and people with mental illness might affect providers’ attitude toward recovery. In addition, the test–retest reliability analysis was conducted in a small sample, and may need to be further examined in future research with a larger number of participants.

We included the introduction describing the concept of recovery [5] in our literal translation of RAQ. Similar to the concept of faith, the concept of recovery also has multifaceted connotations. For example, it often encompasses a traditional meaning of a complete cure. Therefore, the explanation of the meaning of recovery in the RAQ context is a key issue, particularly as the concept of recovery has not yet permeated the mental health field in Japan, and might have resulted in biased responses. The introductory sentences could, for example, provide previously unknown information about recovery, enhance practitioners’ awareness, or even prompt respondents to answer disproportionately based on social expectations. The mean RAQ total score in the present study may therefore be overestimated. Further research is required to examine the appropriateness of the wording used in the introduction.

In Western countries, RAQ has been used for service providers and for people with mental illness [46–52]. Examining its applicability in Japanese people with mental illness may be a relevant topic for future study, particularly as the concept of what constitutes recovery may differ between healthcare providers and people with mental illness [53].

### Limitations

There are some limitations of the present study. First, participants were selected from institutions in specific areas only. In addition, three-quarters of the total study population were registered/assistant nurses, and psychiatric social workers. Although attitudes toward recovery did not differ among occupations in this study, they may vary between areas (urban versus rural), institutional cultures, or professional roles. In short, the generalization of our findings poses a challenge for the future. Second, although the response rate was high, there were a number of non-respondents. The non-responders might have been more likely to report lower attitudes toward recovery and stigma toward people with mental illness. Therefore, the mean RAQ total score in the present study may be slightly overestimated. Further large-scale research that includes a more diverse mental health service provider population is required for robust verification, particularly in terms of the reliability of the Japanese version of the 7-item RAQ.

## Conclusions

The present study demonstrated the factorial validity, concurrent validity, and internal consistency reliability of the Japanese version of the 7-item RAQ among mental health service providers in community and inpatient settings in Japan. We found the Japanese RAQ had acceptable factorial validity, reasonable concurrent validity, and unsatisfactory reliability. Further large-scale research is required to ensure robust verification of the Japanese RAQ.

## Additional file

Additional file 1:
**The Japanese version of the 7-item Recovery Attitudes Questionnaire (RAQ).** (DOCX 31 kb)

## Abbreviations

RAQ: Recovery Attitudes Questionnaire
SDSJ: the Japanese-language version of Social Distance Scale
