# Supplementary material for: Psychometric properties of the Japanese version of the Recovery Attitudes Questionnaire (RAQ) among mental health providers: a questionnaire survey
Source: BMC Psychiatry. 2016 Feb 16;16:32. doi: 10.1186/s12888-016-0740-x (PMC4755029; doi:10.1186/s12888-016-0740-x)
Supplement: Additional file 1: — The Japanese version of the 7-item Recovery Attitudes Questionnaire (RAQ). (DOCX 31 kb) [file 12888_2016_740_MOESM1_ESM.docx]

Appendix 1: The Japanese version of the 7-item Recovery Attitudes Questionnaire (RAQ)

精神の病気をお持ちの方のリカバリーについて、あなたのお考えをお聞かせください。

**次の各文を読んで、あなたのお考えにもっとも近い番号一つに○をつけてください。**

リカバリーは、私たちが皆共有する、過程であり経験です。人は、愛する人の死や離婚、身体の障害や重い精神の病気などを経験した時に、リカバリーという課題にぶつかります。うまくいったリカバリーとは、その経験が起きたという事実や、その影響がまだあるという事実や、また、その人の人生が永遠に変わったという事実を変えることではありません。むしろ、その人が変化し、その人にとっての、それらの出来事の意味もまた変わるということを意味します。その出来事はもはや、その人の人生の一番中心的なことではないのです。(Anthony, 1993)

| No. |  | まったくそう思わない | そう思わない | どちらでもない | そう思う | とてもそう思う |
| --- | --- | --- | --- | --- | --- | --- |
| 1 | リカバリーの過程にある人は、時には後戻りすることもある | 1 | 2 | 3 | 4 | 5 |
| 2 | リカバリーには信念が必要である | 1 | 2 | 3 | 4 | 5 |
| 3 | 精神の病気についての偏見は、リカバリーの進行を遅らせることがある | 1 | 2 | 3 | 4 | 5 |
| 4 | たとえ精神の病気の症状があったとしても、リカバリーは起こり得る | 1 | 2 | 3 | 4 | 5 |
| 5 | 精神の病気の原因を何であると考えていたとしても、リカバリーは可能である | 1 | 2 | 3 | 4 | 5 |
| 6 | 重い精神の病気を持つ人は誰でも、リカバリーするために励むことができる | 1 | 2 | 3 | 4 | 5 |
| 7 | 精神の病気からのリカバリーのしかたは、人によって異なる | 1 | 2 | 3 | 4 | 5 |
